# Supplementary material for: A high-resolution mRNA expression time course of embryonic development in zebrafish
Source: eLife. 2017 Nov 16;6:e30860. doi: 10.7554/eLife.30860 (PMC5690287; doi:10.7554/eLife.30860)
Supplement: Supplementary file 6. [file elife-30860-supp6.zip › biolayout-clusters-files/Cluster155.html]

Cluster155


# Cluster155: Detail

### Go to ZFA detail

## GO

| GO ID | Description | Domain | Annotated | Expected | Observed | Adjusted p-value | Genes | Ensembl IDs |
| --- | --- | --- | --- | --- | --- | --- | --- | --- |
| GO:0007006 | mitochondrial membrane organization | biological\_process | 13 | 0.01 | 2 | 0.0053 | slc25a46 afg3l2 | ENSDARG00000035181 ENSDARG00000062272 |
